# Improving Definition of Screen-Printed Functional Materials for Sensing Application

**DOI:** 10.1021/acsaelm.3c01415

**Published:** 2024-04-05

**Authors:** Lia Campos-Arias, Nikola Peřinka, Yin Cheung Lau, Nelson Castro, Nelson Pereira, Vitor Manuel Gomes Correia, Pedro Costa, José Luis Vilas-Vilela, Senentxu Lanceros-Mendez

**Affiliations:** † BCMaterials, Basque Center for Materials, Applications and Nanostructures, UPV/EHU Science Park, 48940 Leioa, Spain; ‡ Faculty of Science and Engineering, Swansea University, SA1 8EN Swansea, U.K.; § International Iberian Nanotechnology Laboratory (INL), 4715-330 Braga, Portugal; ∥ Physics Centre of Minho and Porto, Universities (CF-UM-UP) and LaPMET - Laboratory of Physics for Materials and Emergent Technologies, University of Minho, 4710-057 Braga, Portugal; ⊥ Centre for MicroElectroMechanics Systems (CMEMS), University of Minho, Campus de Azurém, 4800-058 Guimarães, Portugal; # Grupo de Química Macromolecular (LABQUIMAC) Dpto. Química-Física, Facultad de Ciencia y Tecnología, Universidad del País Vasco (UPV/EHU), Leioa, Bizkaia 48940, Spain; % IKERBASQUE, Basque Foundation for Science, 48009 Bilbao, Spain

**Keywords:** screen-printing, high-end screen, high resolution, stainless-steel stencil, printed electronics

## Abstract

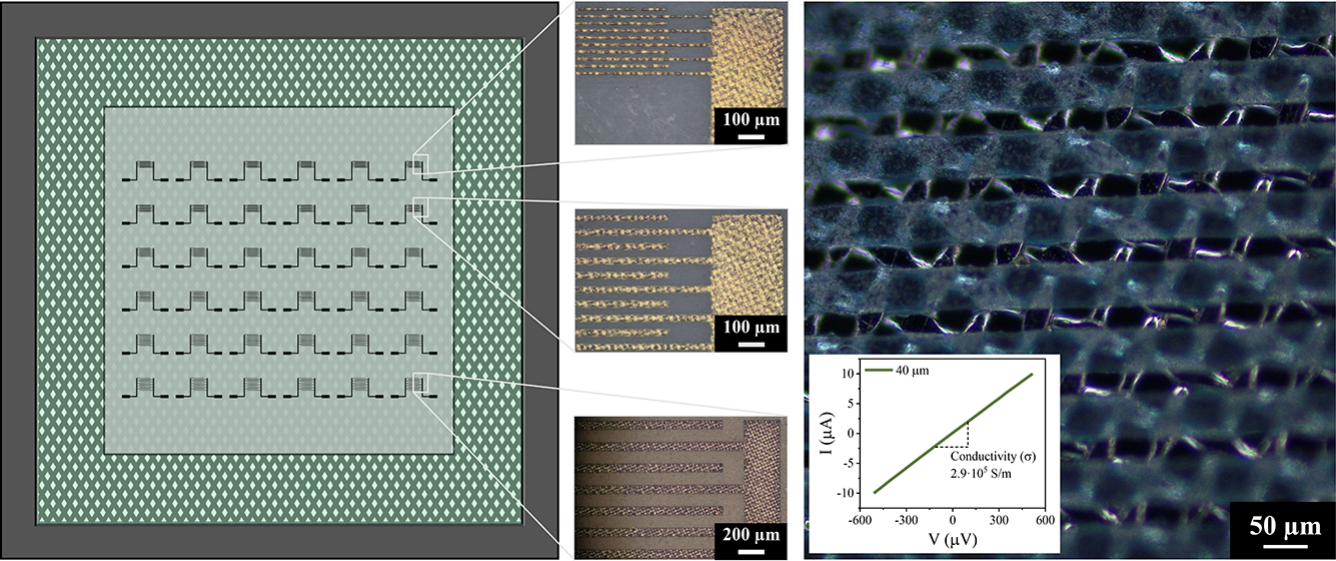

Screen printing is one of the most used techniques for developing printed electronics. It stands out for its simplicity, scalability, and effectivity. Specifically, the manufacturing of hybrid integrated circuits has promoted the development of the technique, and the photovoltaic industry has enhanced the printing process by developing high-performance metallization pastes and high-end screens. In recent years, fine lines of 50 μm or smaller are about to be adopted in mass production, and screen printing has to compete with digital printing techniques such as inkjet printing, which can reach narrower lines. In this sense, this work is focused on testing the printing resolution of a high-performance stainless-steel screen with commercial conductive inks and functional lab-made inks based on reduced graphene oxide using an interdigitated structure. We achieved electrically conductive functional patterns with a minimum printing resolution of 40 μm for all inks.

## 1 Introduction

Integrated smart systems have been key in the development of printed electronics. This field is widely explored for fabricating antennas^1^ and smart tags^2,3^ that can be used in food industry^4^ and medication control,^5^ among others. In other fields such as sport fitness or healthcare monitoring, where wearable devices are required for detecting variations in blood pressure, temperature, or specific biomolecules, printing technologies have been also used because they allow the fabrication of lightweight, thin, and conformable electronics.^6^

As one of the most used methods for printed electronics, screen printing stands out for its simplicity, scalability, effectivity, and robustness.^7,8^ This leading technique allows the combination of multiple layers of different materials onto a flexible substrate by transferring the ink through a mesh with a blade/squeegee. By screen printing or by combining this method with other printing methods, different sensing devices can be fabricated, including environmental sensors (temperature,^9^ humidity,^10^ gas detection^11^), biosensors (glucose,^12^ blood pressure^13^), pressure sensors (touch sensors^14^), and light sensors.^15^ Many of them are based on a simple structure which consists on a substrate, interdigitated electrodes, and the sensing film.^6,16^

For sensing applications based on conductometric measurements, planar interdigitated electrode arrays are one of the most used structures.^17,18^ These structures benefit from having a large contact area with the sensing material, show design flexibility, reduced sizes, and affordable prices.^19−21^

In the photovoltaic (PV) industry, screen printing is the predominant method used to metallized crystalline silicon (c-Si) solar cells.^22^ The precision of the line grids printing in this process is essential for improving performance of the solar cells, and, in this way, this industry has pushed technology to achieve high performance metallization pastes and high-end screens. As a result, the printed lines have been reduced in size, being the minimum contact finger width (*w*_f_) approximately 25 μm at the industrial scale and ca. 20 μm at the lab scale in 2022.^23^

In other applications, such as organic electrochemical transistors (OECT), where competing printing techniques can reach thinner lines (e.g., inkjet printing or aerosol jet printing), screen printing has not been fully exploited. For instance, the limiting factor in an OECT is the channel length, and the range achievable by screen printing oscillates between 80 and 200 μm.^24−26^ Its low resolution compared with digital printing precludes the manufacture of miniature circuits with high precision by this means. This feature is affected by ink rheology, printer hardware (screen, squeegee, and substrate), and the printing process (printing speed, pressure, and snap-off distance).^27^

**Figure 1:**
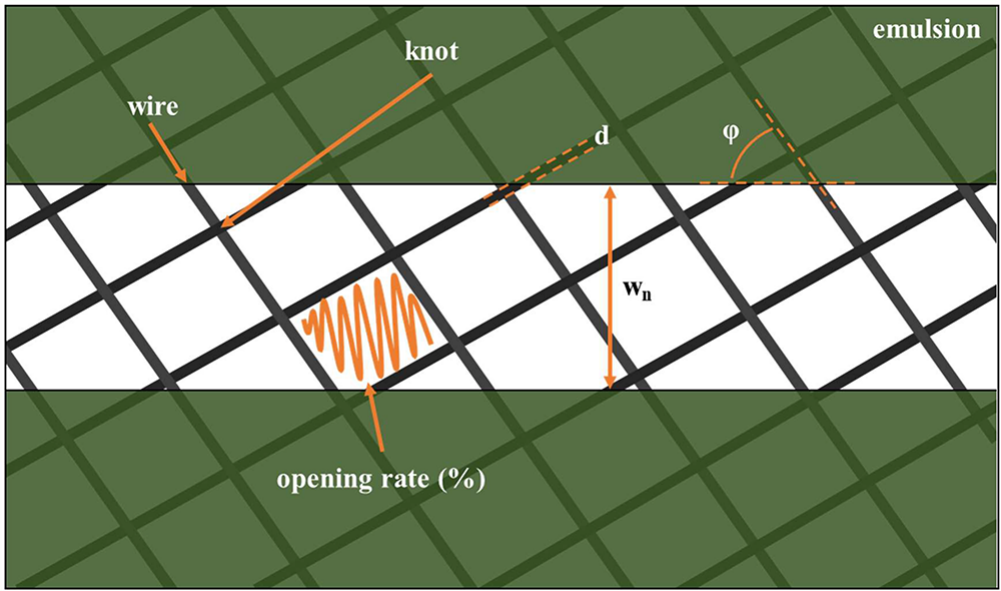
Schematic representation of part of a screen with its main characteristic parameters.

Focusing on the printer hardware, the quality of the finger geometry is directly related to the quality of the screen, which in turn depends on the mesh and the emulsion applied on it. Parameters that define the former are the mesh count (MC) and the wire diameter (*d*), while the latter is defined by the nominal screen opening width (*w*_n_) and the angle between emulsion edge and mesh wires (φ).^28^Figure 1 shows a schematic representation of part of a screen.

There are some challenges of the screen-printing process that gain significance when printing fine lines, such as disruptions of the finger geometry, finger interruptions, or excessive spreading.^29^ In order to obtain better quality screens, there have been developed fine meshes with increasing MC and decreasing wire thickness (*d*_w_). Another way to enhance the resolution is the use of a “knotless screen”. This is a screen with an angle of 0° (state-of-the-art screens have an angle of 22.5°) with respect to the fingers of the grip, which means that between two adjacent wires there is a finger opening. In this way, mesh marks are effectively reduced because there are no wire intersections in the prints. However, these screens are quite sophisticated to fabricate due to the difficulty of aligning the finger openings between the wires.^23^

Another feature of the screen that affects the printing resolution is the thickness of the emulsion over the mesh (EOM). As explained in ref (30), there is a transfer limit between emulsion opening width and height. Increasing the EOM thickness reduces the finger width and increases its thickness; nonetheless, this trend reverses for narrower fingers. Thus, it is necessary to reduce the EOM thickness to increase the resolution of the fingers.

In this context, significant progress in the development of stainless-steel screens has been achieved. Meshes woven with higher tensile strength wires than standard meshes have been manufactured to cope with screen printing demands such as high dimensional accuracy, screen mask longevity, and fine line printing (30 μm electrode lines).^31^ In this work we have used one of those stainless-steel screens that has also been roll-calendered to reduce its thickness and roughness, thus improving the print laydown and resolution.^31^ Moreover, the EOM thickness is only 5 μm. Here, we optimize and compare printing resolution of different commercial and functional lab-made^32^ inks screen-printed in an interdigitated (ID) structure, with a high-performance stainless-steel screen.

## 2 Experimental Section

### 2.1 Materials

Different commercial inks were purchased from several sources: Orgacon SI-P2000 (nanosilver in 2-butoxyethanol, pentanoic acid, 1-methoxy-2-propanol, and 2-pyrrolidone screen printing ink, 65–70% solid content, density 2.26 g·cm^−3^, viscosity (AR2000-rheometer, 25 °C, 100 s^–1^): 5.5 Pa·s, sheet resistance <3 mΩ/sq/25 μm, AGFA, Belgium), DuPont PE827 (silver–copper ink in triethyl phosphate, 76–80% solid content, density 2.6 g·cm^−3^, viscosity (Brookfield RVT, #14 spindle, 10 rpm, 25 °C): 15–50 Pa·s, sheet resistance <60 mΩ/sq/25 μm, DuPont Ltd., UK), SCAG-004P (silver in 2-(2-butoxyethoxy)ethanol, diethyl sebacate, and epoxy resin, 70–80% solid content, density 3.1 g·cm^−3^, viscosity (rheometer, 23 °C, 10 s^–1^): 38.9 Pa·s, sheet resistance <60 mΩ/sq/25 μm, Mateprincs, Spain), and Clevios SV4 STAB (1.5 wt % PEDOT:PSS screen-printable dispersion in 2,2′-oxyethanol and [3-(2,3-epoxypropoxy)propyl]diethoxymethylsilane, viscosity (Brookfield DV2T, CPA-52Z spindle, 10 s^–1^, room temperature): 10.8 Pa·s, sheet resistance 500 Ω/sq, Heraeus, Germany). As for the inks prepared in the laboratory, they were based on conducting graphene oxide; reduced graphene oxide (rGO) FP200 1100 (bulk density 4.6 × 10^–2^–5.2 × 10^–2^ g·cm^–3^, specific surface area 300–350 m^2^·g^–1^, conductivity 7 S·cm^–1^) and nitrogen-doped rGO (NrGO) 11-1100 (bulk density 5 × 10^–2^–6 × 10^–2^ g·cm^–3^, specific surface area 190–220 m^2^·g^–1^, conductivity 3.3 S·cm^–1^) and were provided by Abalonyx AS, Norway. Calprene H6180X SEBS polymer (styrene–ethylene–butylene–styrene, 85% ethylene–butylene, 15% styrene) and Calprene H6120 SEBS polymer (68% ethylene–butylene, 32% styrene) were supplied by Dynasol Gestión S.A., Spain. *p*-Cymene (1-isopropyl-4-methylbenzene, 99%) was purchased from Sigma-Aldrich.

The substrates used were Flextrace T24 (thickness 125 μm, roughness 5 μm, surface energy 65.0 ± 0.7 mJ·m^−2^) and PET Melinex ST506 (thickness 125 μm, roughness 0.00153 μm, surface energy 39.0 ± 1.0 mJ·m^−2^) and were purchased from IKONICS Corporation, USA, and from SABIC Snij-Unie, The Netherlands, respectively. Screen MS640/15CL17 for screen printing was provided by Asada Mesh Co., LTD, Japan. The parameters of the screen were: 640 wires per linear inch, 15 μm wire diameter, trampoline configuration screen frame size 320 × 320 mm^2^ (22.5°) (inner size 220 × 220 mm^2^), 17 μm thickness (calendered), a tension of 26 N·cm^–1^, *R*_*z*_ = 2.46 μm, snap off = 1 mm, 39% opening rate, 5 μm emulsion over mesh (EOM).

### 2.2 Lab-Made Inks Preparation

Inks were prepared by sonicating (ATU, ATM3L) the filler in *p*-cymene until it was well dispersed; SEBS was added afterward, and the mixture was stirred (IKA, RH D S000) for 1–2 h until the polymer was completely dissolved. Then, each ink was screen-printed (semiautomatic DX3050P, Shenzhen Dstar Machine Co.) in both tested substrates with the following parameters: printing rate of ∼0.1 m·s^−1^ (∼0.4 m·s^−1^ for the ink Orgacon SI-P2000), printing pressure of ∼25 N over 120 mm of a squeegee length, and a printing squeegee angle of 70°. Then, samples were cured (J.P. Selecta, Digitronic-TFT) at 100 °C for 30 min. Commercial inks were also screen-printed with the same parameters and cured at 120 °C for 30 min. In Table 1 it is summarized the information of the lab-made inks.

**Table 1:** Composition of the Inks Prepared in the Lab

	filler (wt %)	polymer (wt %)	SEBS (g):*p*-cymene (mL)
SEBS/15%rGO	15	85 (H6180X)	1:7
SEBS/15%NrGO	15	85 (H6120)	1:4

### 2.3 Characterization

Previous to analyzing the printability of the inks, the rheological behavior of the lab-made inks was evaluated at room temperature with a Brookfield DV2T touch screen viscosimeter using a CPA-52Z cone–plate (3° cone angle, 1.2 cm cone radius).

**Figure 2:**
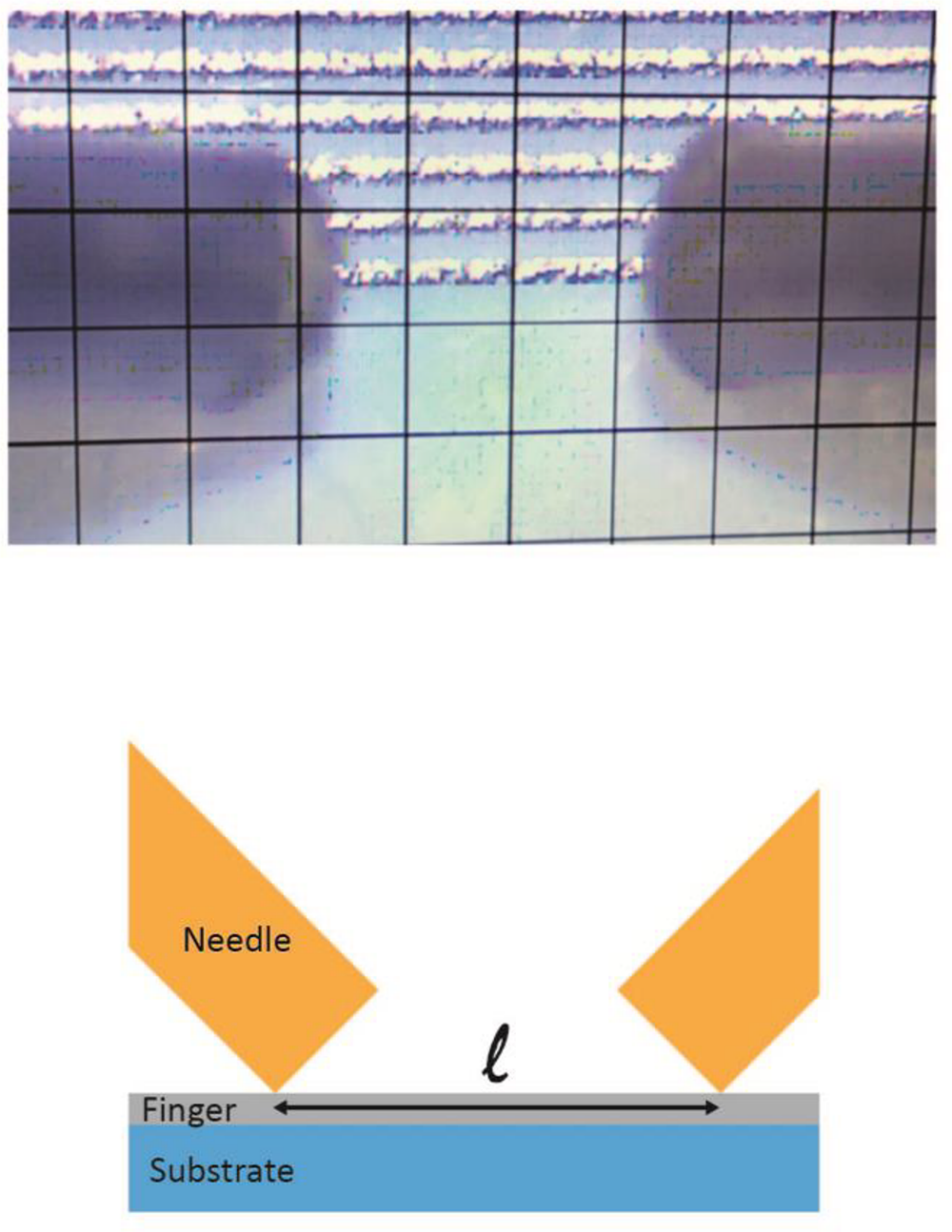
Image of the IV probe needles and a schematic representation of the measurement.

The morphology of the printing patterns was analyzed using an optical microscope (Olympus BX53MRF-S). Topographical data (thickness and roughness) were obtained by profilometry (KLA Tencor D-100), scanning the fingers with a stylus force of 1 mg (stylus radius 2 μm, rate 0.05 mm·s^−1^). Finally, the electrical conductivity of the samples was measured with a Keithley 2400 source meter unit, measuring the samples directly on the printed fingers using a precision manual multidimensional probe station with IV probe needles (Figure 2). The use of a two-probe configuration was necessary to estimate the conductivity of the samples due to the dimensional constraints imposed by the dimensions of the fingers. The penetration of the printed ink by the pointer section of the needle results in a cut of the finger. In order to mitigate this phenomenon in the measuring process, the broader section of the needle was employed, thus allowing a greater surface area of contact and reducing the extent of penetration.

## 3 Results and Discussion

### 3.1 Lab-Made Inks Rheology

**Figure 3:**
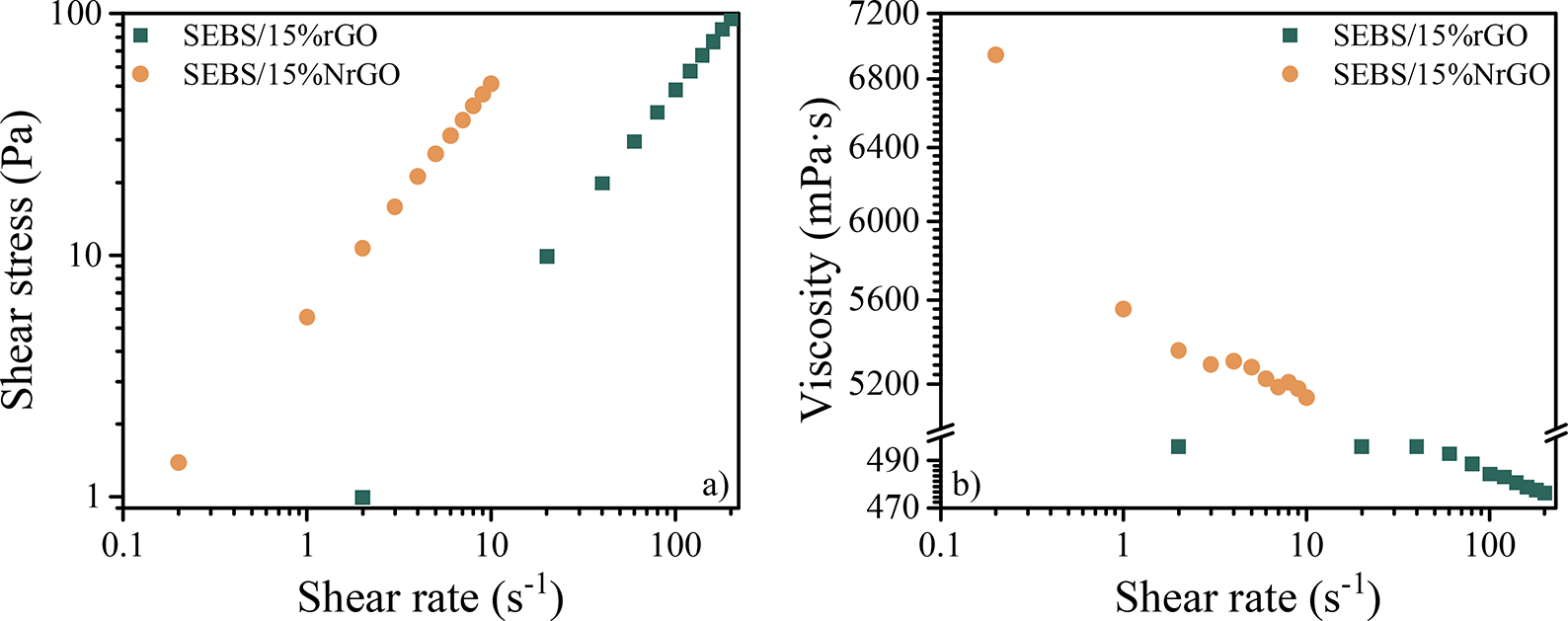
Inks rheological characteristics: variation of shear stress (a) and viscosity (b) as a function of shear rate on a logarithmic scale.

Figure 3 shows the rheological characteristics of the inks. Both inks present a non-Newtonian fluid behavior in which the viscosity decreases with increasing shear rate. This effect is more noticeable for ink with NrGO which shows an adequate viscosity for screen printing while rGO ink viscosity is below the optimal range^33^ (1000–10000 mPa·s). The difference in viscosity is due to the solvent content: SEBS/15%NrGO has approximately half the volume of solvent than SEBS/15%rGO because NrGO is better dispersed in *p*-cymene than rGO. Nonetheless, both inks were successfully printed by screen printing.

### 3.2 Morphological Analysis

**Figure 4:**
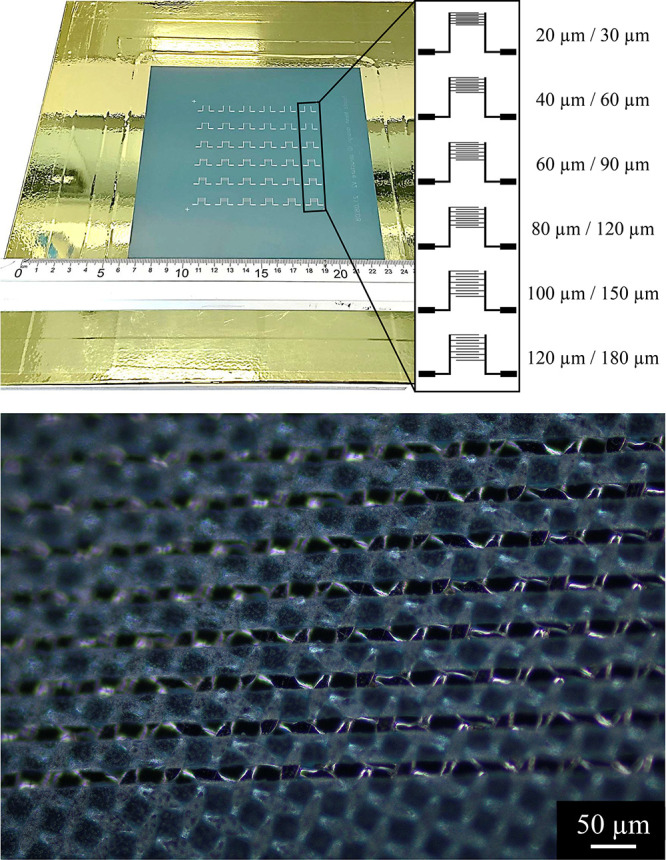
Top: picture of the screen and an enlargement of the ID electrodes with the dimensions of the fingers. Bottom: optical microscopy image of the 20 μm fingers of the ID electrode.

The used screen had six rows of interdigitated (ID) electrodes with different finger widths and distances between them as it is shown in Figure 4 in the format: width/distance. Interdigitated electrodes are relevant in sensing technologies due to its ease and low cost of fabrication as well as its high sensitivity. For sensing applications, the distance between fingers is related to the data rate capability: the smaller the distance, the faster the data rate.^34^ Thus, it is important to reduce the distance in ID electrodes in order to increase the electric field strength. Further, reducing the width of the fingers allows the probing electric field to be restricted to the sensing space.^35−39^

**Figure 5:**
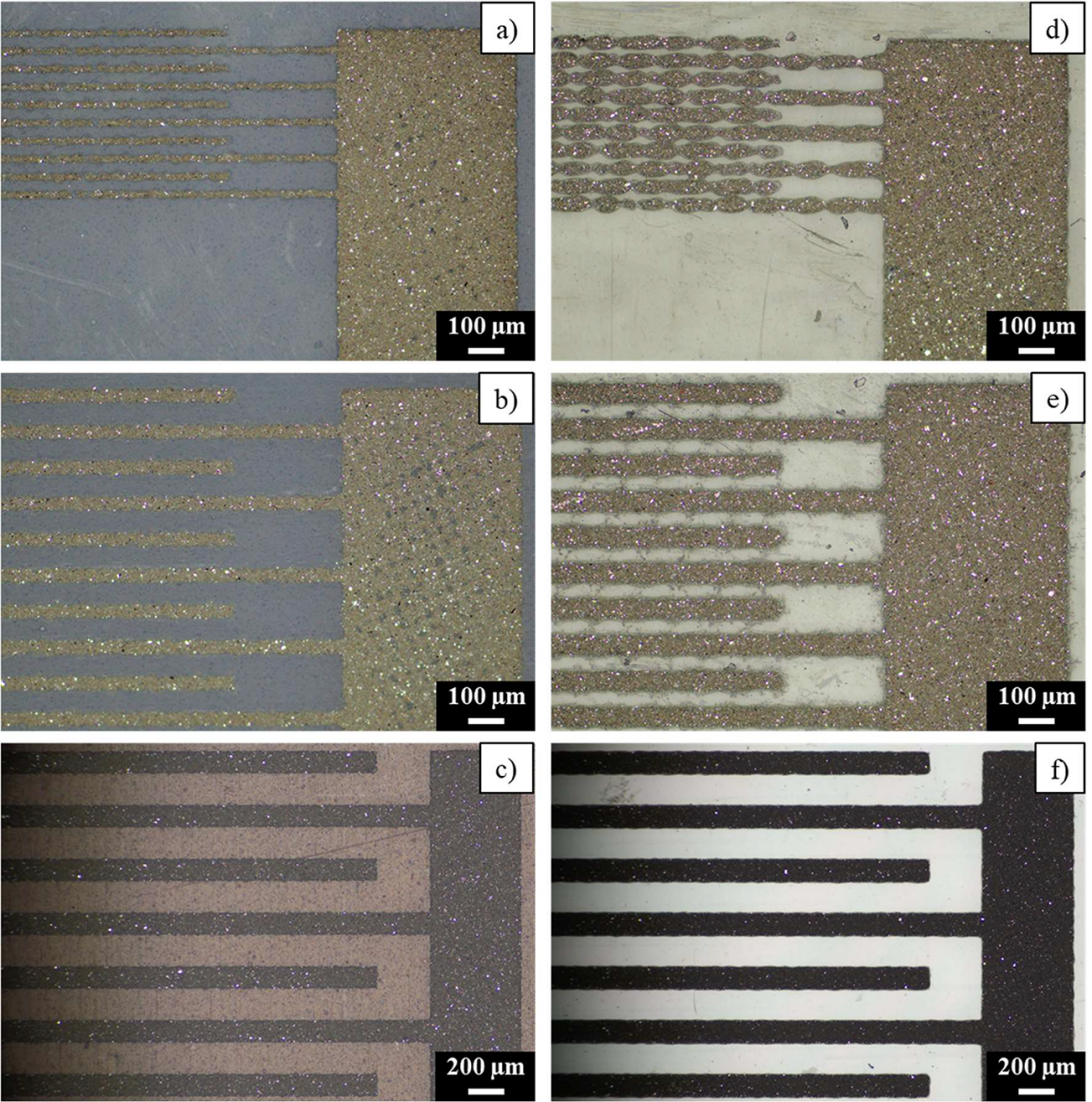
DuPont PE827 prints with different finger widths: (a) 20, (b) 40, and (c) 120 μm on Flextrace; (d) 20, (e) 40, and (f) 120 μm on Melinex.

The printed samples were observed by optical microscopy to determine the minimal printable finger width and lateral distance. Figure 5 displays the images obtained for DuPont PE827 ink printed on Flextrace and Melinex (the same information for the other inks is presented in the Supporting Information). It was found that every ink has a satisfactory printability up to 40 μm finger width with a distance of 60 μm between fingers. The homogeneous and continuous fingers of the printed inks are effective, and their definition is good. When the finger width is 20 μm, the line loses continuity. In some of the prints, the texture of the mesh is also visible (Figure S1). Electrodes printed on Flextrace have a better definition than electrodes printed on Melinex because the higher surface energy of Flextrace allows the ink to wet more easily.^40^ Moreover, the higher roughness of Flextrace seems to play an important role in the enhancement of the adhesion of the ink to the substrate. In the images of the traces printed on Melinex, it is observed that the interdigitates are formed by drops joined by thinner lines. It is more visible when the interdigitates are thinner and closer to each other. For example, in Figure 5d fingers are wider than the space between them, even making contact among fingers in some points. This is confirmed by profilometry measurements explained in the following section.

**Figure 6:**
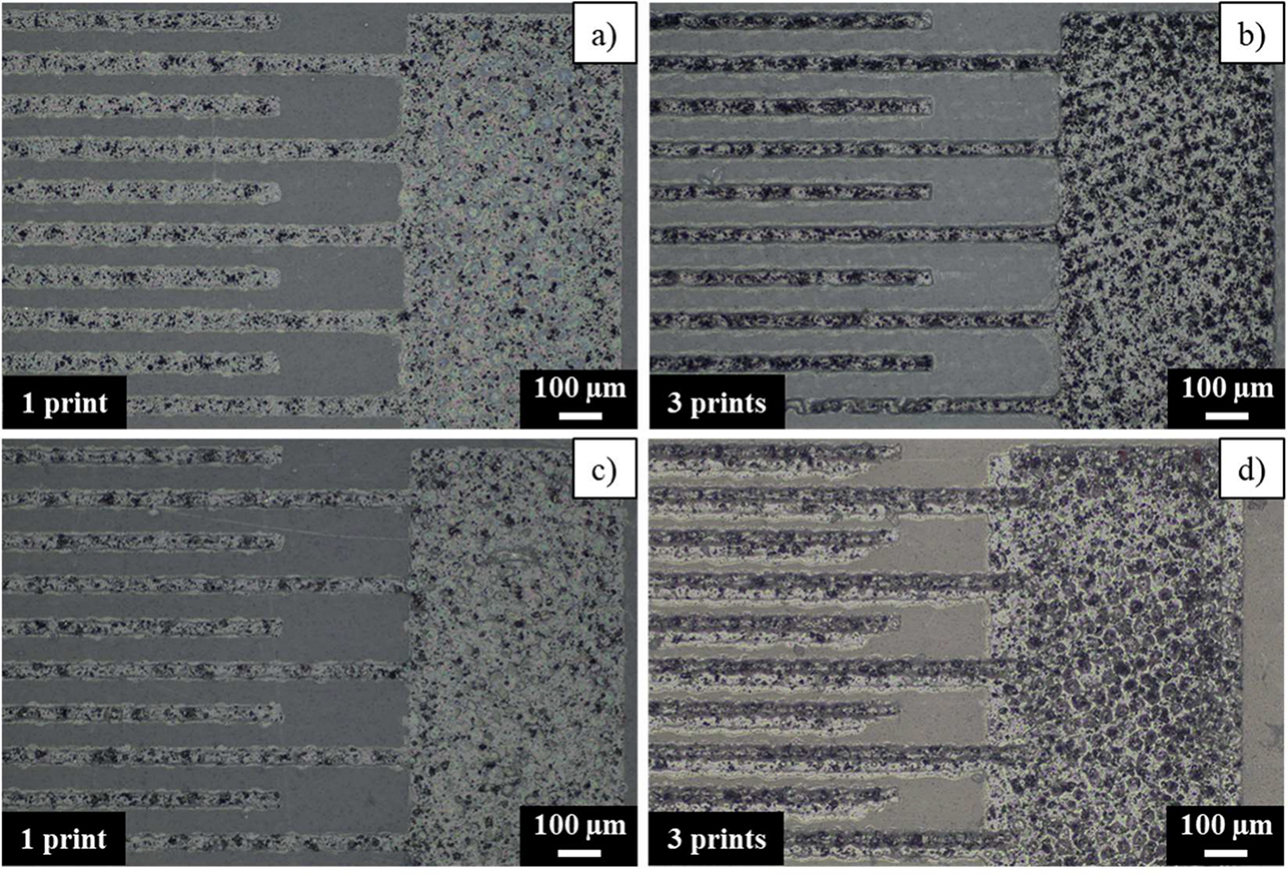
Lab-made inks screen printed on Flextrace: SEBS/15%rGO (a) 1 and (b) 3 prints; SEBS/15%NrGO (c) 1 and (d) 3 prints.

In order to analyze the difference between commercial and lab-made inks, Figure 6 displays 40 μm fingerprints of lab-made conductive inks. First of all, it was necessary to print more than one layer to cover the whole area. The main problem of printing several layers is the use of a high-resolution screen, challenging the printing position repeatability of the used semiautomatic screen-printing machine. Moving the substrate slightly triggers a noticeable misalignment between layers, even printing wet on wet, as can be observed in Figure 6d. Regarding homogeneity, the matrix and filler can be distinguished for SEBS/15%rGO and SEBS/15%NrGO inks. The reason might be that the sizes of rGO and NrGO agglomerates are bigger than the mesh design, and they cannot get through the mesh structure completely, presenting a lower content of filler in the final print.

### 3.3 Profilometry

Since the minimum continuous finger width achieved for all inks was 40 μm, profilometry analysis was performed with those electrodes.

Table 2 summarizes the finger widths of the samples obtained by profilometry and by ImageJ software. Generally, finger width is closer to the nominal value when printed on Flextrace than on Melinex, upholding the microscopy results. Clevios SV4 STAB printed on Flextrace could not be measured by profilometry because the thickness of the sample was in the range of the substrate roughness. Something to consider is that comparing the width measurements obtained by profilometry and ImageJ software, the former presents smaller values than the latter in almost every case. The reason for this lies in the sensitivity of the profilometry technique which cannot identify the lateral start and ending point of the lines properly, as prints are too thin in the edges. Thus, those measurements have an associated default error.

**Table 2:** Finger Widths Measured by Profilometry and ImageJ Software of 40 μm Nominal Finger Width Samples and the Corresponding Viscosity of the Inks^a^

	width (μm)		
ink	profilometry	ImageJ	viscosity (Pa·s)
AGFA SI-P2000 F	42 ± 4	49 ± 2	5.5
AGFA SI-P2000 M	39 ± 6	42 ± 2	
DuPont PE827 F	46 ± 4	44 ± 3	15–50
DuPont PE827 M	62 ± 4	66 ± 3	
SCAG-004P F	40 ± 3	47 ± 2	38.9
SCAG-004P M	60 ± 6	72 ± 3	
SV4 STAB F		53 ± 2	10.8
SV4 STAB M	44 ± 2	46 ± 3	
SEBS/15%rGO F 1x	47 ± 11	55 ± 4	0.484
SEBS/15%rGO F 3x	38 ± 4	64 ± 8	
SEBS/15%NrGO F 1x	45 ± 8	49 ± 3	5.14
SEBS/15%NrGO F 3x	48 ± 5	83 ± 4	

a F is for samples printed on Flextrace and M for samples printed on Melinex. Measurements are an average of 10 fingers for each electrode.

**Figure 7:**
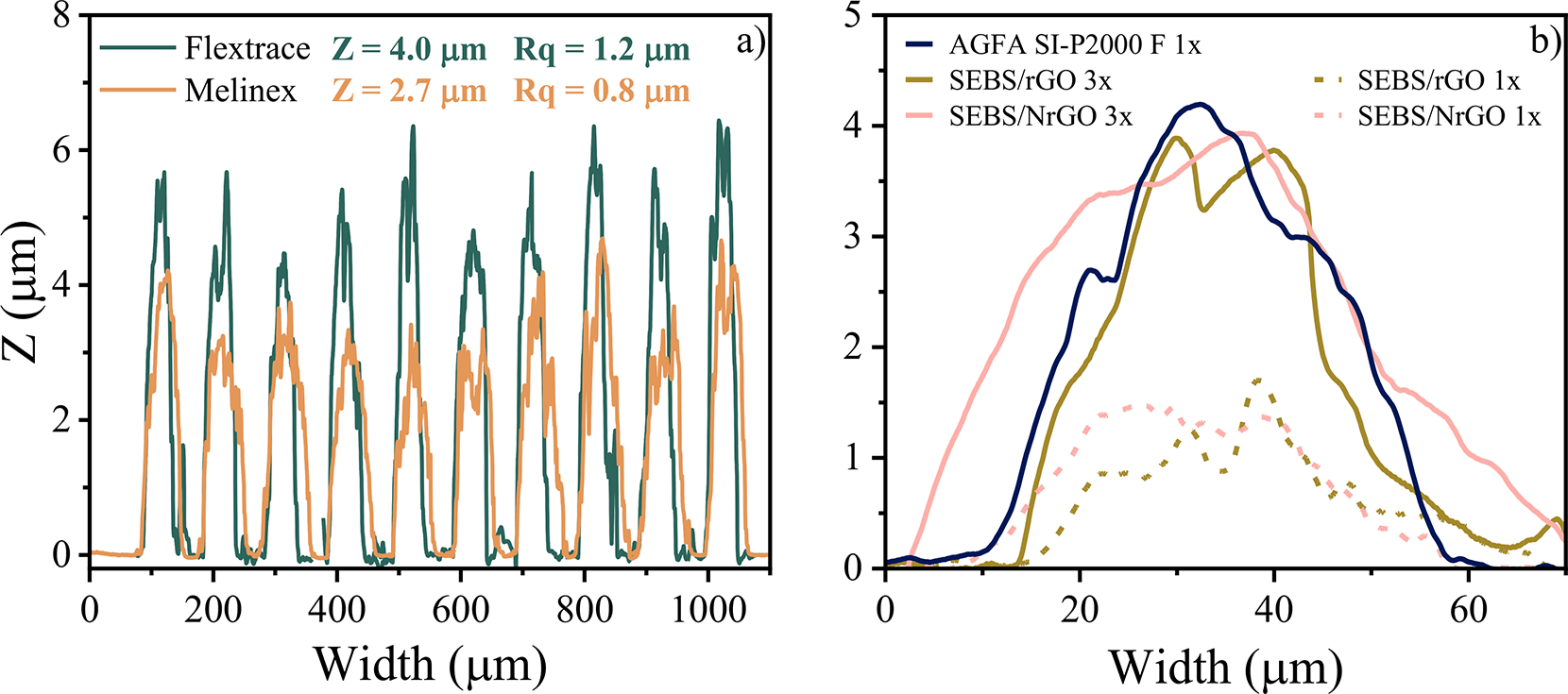
(a) Finger thickness of DuPont PE827 ink printed on Flextrace and Melinex. (b) 40 μm fingers of lab-made inks with one and several layers and AGFA SI-P2000 ink.

In Figure 7a and Figure S4, the finger thickness and rugosity of the inks printed on both substrates are compared. Since printing on Melinex generates wider electrode fingers, due to the lower surface energy of the substrate, it results in having less thickness than printing on Flextrace. In the case of lab-made inks, thickness was lower than commercial inks when only one layer is printed, and as is shown in the optical microscopy images, the area was not fully covered. For that reason, it was necessary to print 3 layers of the conductive lab-made inks.

The difference in thickness between commercial inks and lab-made inks, considering that all have a contact angle lower than 10°, is a consequence of the viscosity. Lab-made inks present lower viscosity than commercial inks, triggering a major spreading of the ink once deposited onto the substrate. In Figure 7b it is displayed the profilometry results of a 40 μm finger lab-made inks of 1 and 3 layers and a 40 μm finger of a commercial ink to compare the difference in thickness and width. A summary of thickness values of all of the samples is displayed in Table S1.

Compared with other electrodes fabricated by this method, the lowest finger width obtained with this screen is lower than ID electrodes found in the literature, as is observed in Table 3. Nonetheless, compared with other applications, such as photovoltaic industry, the lateral resolution achieved is lower than 40 μm.

**Table 3:** Finger Dimensions of ID Electrodes Found in the Literature

ID electrode material	finger width (μm)	space between fingers (μm)
silver^21^	200	2500
gold^20^	100	100
silver^10^	520–560	1500
silver^14^	750	750

### 3.4 Electrical Conductivity

**Figure 8:**
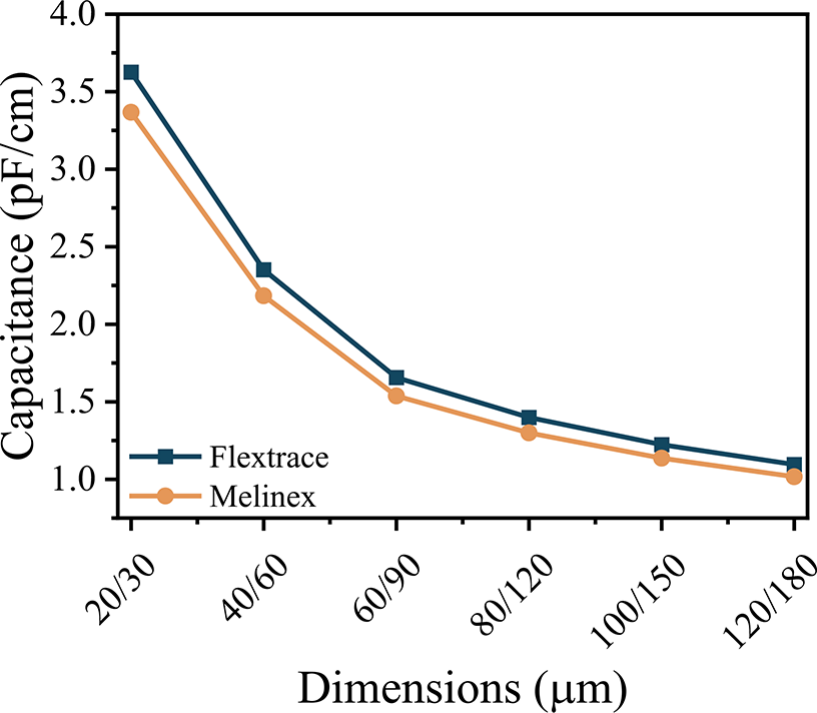
Theoretical calculation of capacitance in function of area for interdigital designs using a Flextrace and Melinex substrate.

First, the capacitance of the ID electrodes has been calculated theoretically, and it is represented in Figure 8 as a function of the ID electrode dimensions. The capacitance formed by an interdigitated capacitor depends on the dielectric constants of the substrate and its thickness as well as the surrounding medium where it is placed, in this case air. Thus, the fringing capacitance and transverse capacitance also depend on the geometric characteristics of the fingers and their spatial arrangement (electrode width, electrode thickness, distance between electrodes and length), these parameters being defined in Table 4.

**Table 4:** Interdigital and Substrate Parameters

	Flextrace	Melinex
substrate dielectric constant	3.2	2.9
substrate thickness	125 × 10^–6^ m	
number of fingers	10	
finger length	3.7 × 10^–3^ m	

According to the literature,^41,42^ calculations of the capacitance based on the geometry of the interdigital designs allow to tune the capacitance value as a function of the dielectric constant of the substrates. As can be observed in Figure 8, the capacitance increases with the increase in the number of fingers by area unit. As the spacing between fingers increases, the effective area for the number of fingers decreases, leading to a decrease in the capacitance as a function of the total area. Also, the dielectric constant of the substrate plays a role in the capacitance, increasing the capacitance with the dielectric constant.

**Figure 9:**
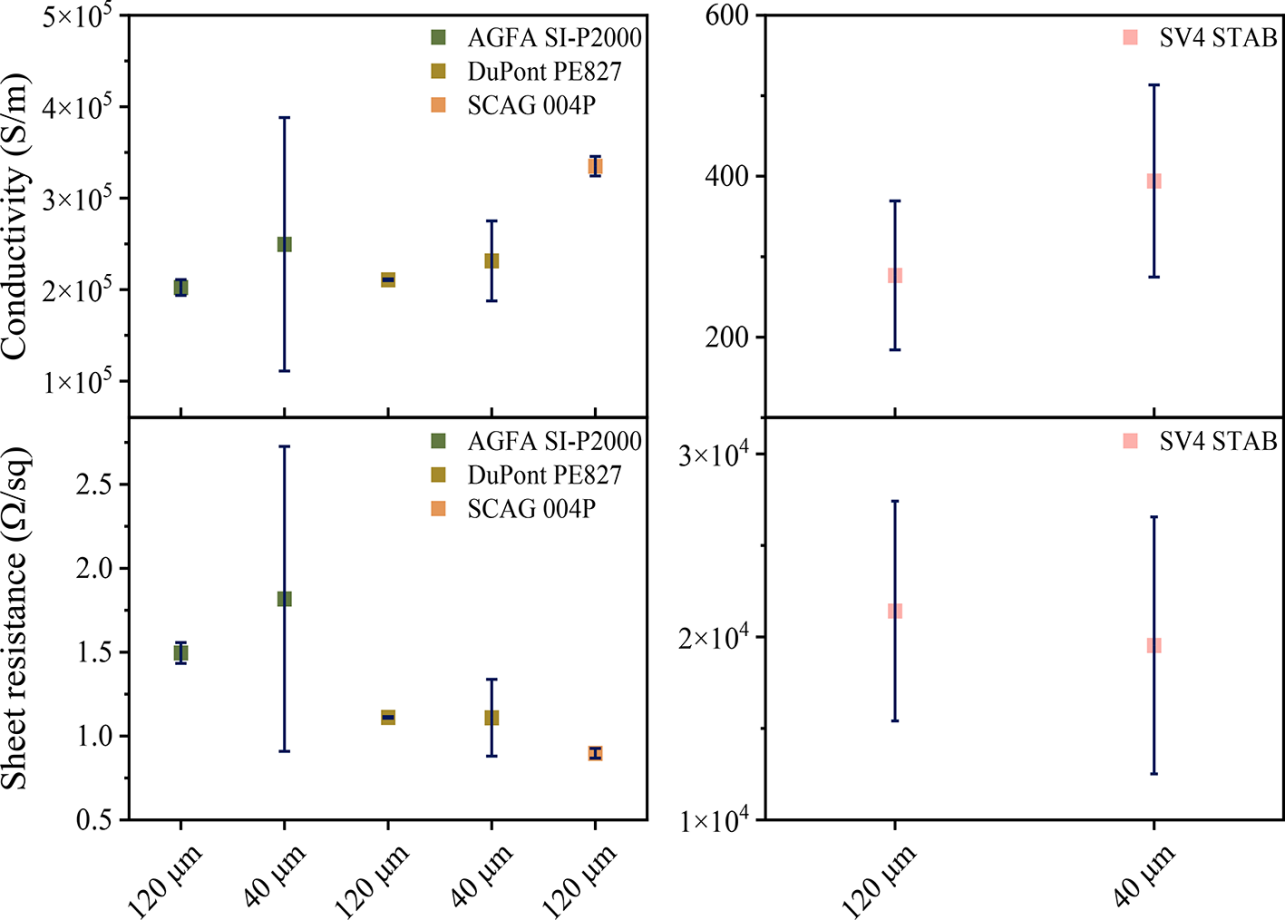
Electrical conductivity and sheet resistance of commercial inks printed on Flextrace.

In order to evaluate if the inks are functional as interdigitated electrodes and considering the better print quality for the maximum resolution, the electrical conductivity of the commercial inks was measured. For a proper comparison, the 120 and 40 μm finger width samples were used, as displayed in Figure 9. It is observed that measurements of 120 μm present lower standard deviation than measurements of 40 μm width, as expected since lines are better defined and the area is fully covered. The electrical conductivity of the 40 μm finger width SCAG-004P sample corresponds to insulator values, meaning that even if the print looked acceptable there were some discontinuities.

## 4 Conclusions

High-resolution screen printing plays a pivotal role in advancing the field of printed electronics by overcoming several limitations associated with conventional screen-printing methods. This technology effectively addresses a range of challenges, including, but not limited to: (i) facilitating the miniaturization of electronic devices and sensors, essential for the fabrication of sensor arrays and portable devices; (ii) enabling the creation of micropatterns for transparent functional surfaces and sensor structures; (iii) enhancing the sensitivity of planar sensors utilizing interdigitated topologies, attributed to an increased interface between electrodes and sensing materials; (iv) mitigating crosstalk and achieving higher track density in multilayer or intricate printed circuits; and (v) improving the reproducibility of printed devices and sensors through enhanced printing resolution. With regard to the impact on the screen printing process parameters, high-resolution structures can be achieved by the use of stainless steel meshes with high mesh counts.

In this work, high-resolution ID structures fabricated using different functional inks were successfully printed with sub-120 μm resolution using a high-definition stainless steel screen. Samples of 40, 60, 80, 100, and 120 μm line width were continuous and presented widths close to the nominal width. The nominal line width increased more significantly on the Melinex ST506 substrate than on Flextrace T24 due to the lower surface energy of the first one, resulting in prints with lower definition. Regarding the new functional inks based on rGO and NrGO, they required repetitive prints to achieve the printed layer continuity and thickness of the commercial ones since graphene forms agglomerates bigger than the opening rate of the mesh. Thus, it would be beneficial to functionalize the filler in order to enhance the dispersion within the solvent. Theoretical capacitance calculus indicates that the lower the space between the fingers, the higher the capacitance values. Finally, conductivity measurements reveal that 40 μm line width ID electrodes have slightly higher sheet resistance than 120 μm line width ID electrodes, but values are in the same order of magnitude which enables them to work as electrodes in sensing applications. Thus, the present work represents a relevant contribution regarding the increase in the resolution achieved in the development of screen-printed ID electrodes for sensing applications.

## Supporting Information Available

Additional images of printed samples AGFA SI-P2000, SCAG-004P, and SV4 STAB; finger thickness and roughness of all samples (PDF)
