# Supplementary material for: Improving Definition of Screen-Printed Functional Materials for Sensing Application
Source: ACS Appl Electron Mater. 2024 Apr 5;6(4):2152–60. doi: 10.1021/acsaelm.3c01415 (PMC11044814; doi:10.1021/acsaelm.3c01415)
Supplement: Supplementary file 1 — el3c01415_si_001.pdf [file el3c01415_si_001.pdf]

Supporting Information: Improving Definition of Screen-printed Functional Materials for Sensing Application

Lia Campos-Arias[a], Nikola Peřinka[a], Yin Cheung Lau[b], Nelson Castro[c,d], Nelson Pereira[d], Vitor Manuel Gomes Correia[e], Pedro Costa[d], José Luis Vilas-Vilela[a,f] and Senentxu Lanceros-Mendez[a,g]*

[a] BCMaterials, Basque Center for Materials, Applications and Nanostructures, UPV/EHU Science Park, 48940 Leioa, Spain

[b] Faculty of Science and Engineering, Swansea University, SA1 8EN Swansea, UK

[c] International Iberian Nanotechnology Laboratory (INL), 4715-330 Braga, Portugal

[d] Physics Centre of Minho and Porto, Universities (CF-UM-UP) and LaPMET - Laboratory of Physics for Materials and Emergent Technologies, University of Minho, 4710-057, Braga, Portugal

[e] Centre for MicroElectroMechanics Systems (CMEMS), University of Minho, Campus de Azurém, 4800-058, Guimarães, Portugal

[f] Grupo de Química Macromolecular (LABQUIMAC) Dpto. Química-Física, Facultad de Ciencia y Tecnología, Universidad del País Vasco (UPV/EHU), Leioa, Bizkaia 48940, Spain

[g] IKERBASQUE, Basque Foundation for Science, 48009 Bilbao, Spain

[*] Corresponding author. E-mail address: lia.campos@bcmaterials.net

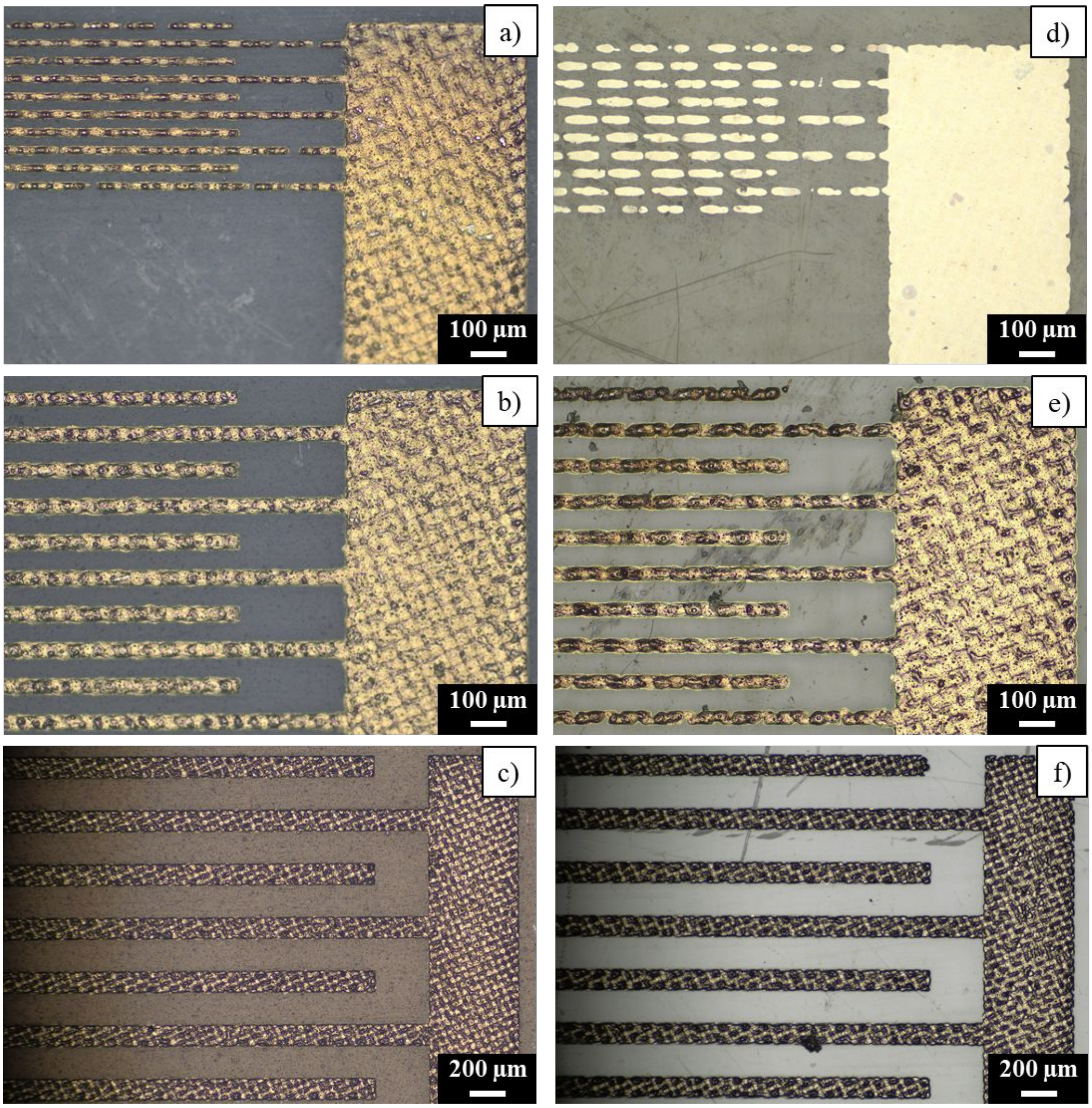

Figure S1. Optical microscope images of AGFA SI-P2000 prints with different finger widths: a) 20 µm, b) 40 µm, and c) 120 µm on Flextrace, and d) 20 µm, e) 40 µm, and f) 120 µm on Melinex.

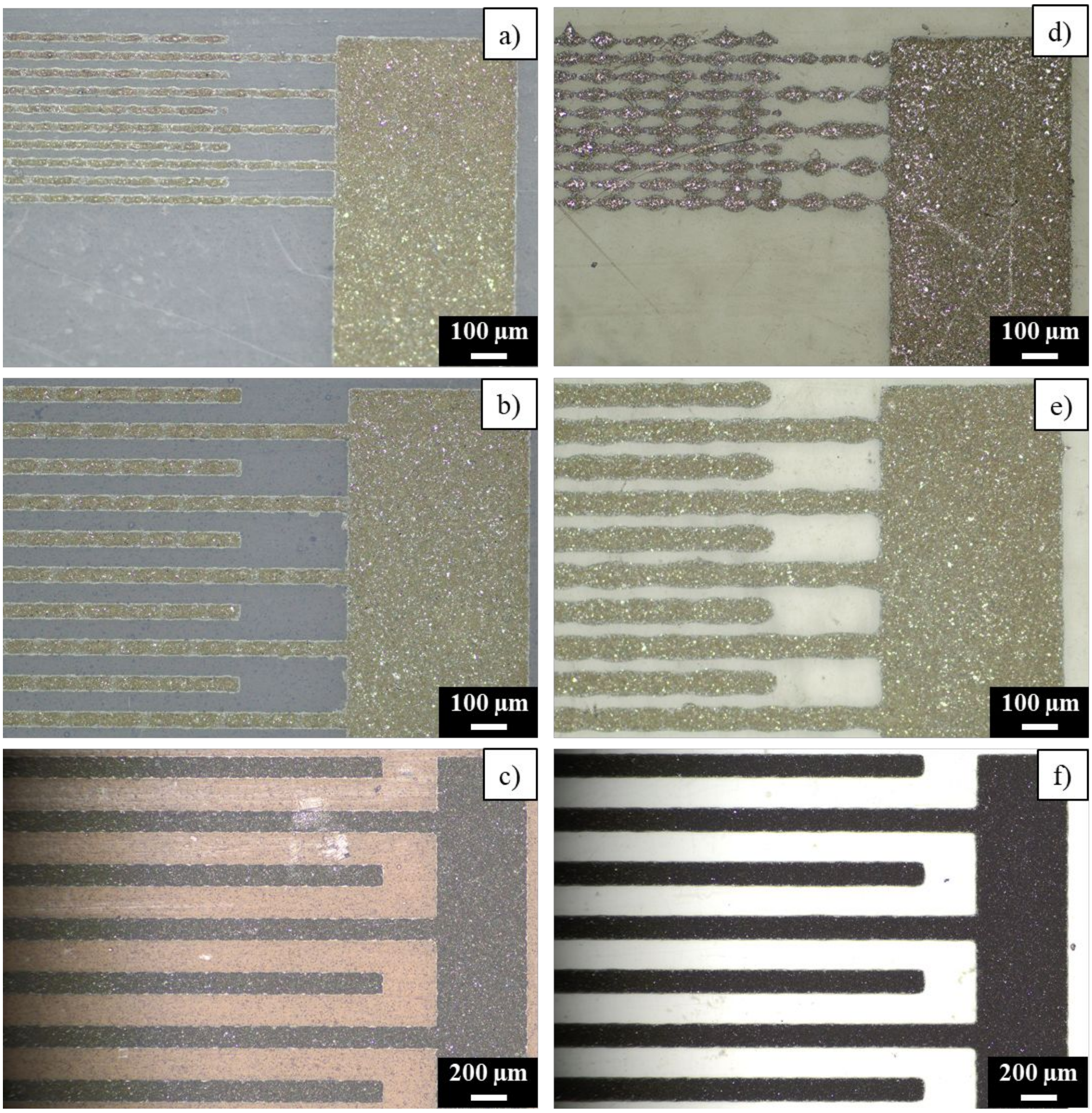

Figure S2. Optical microscope images of SCAG-004P prints with different finger widths: a) 20 µm, b) 40 µm, and c) 120 µm on Flextrace, and d) 20 µm, e) 40 µm, and f) 120 µm on Melinex.

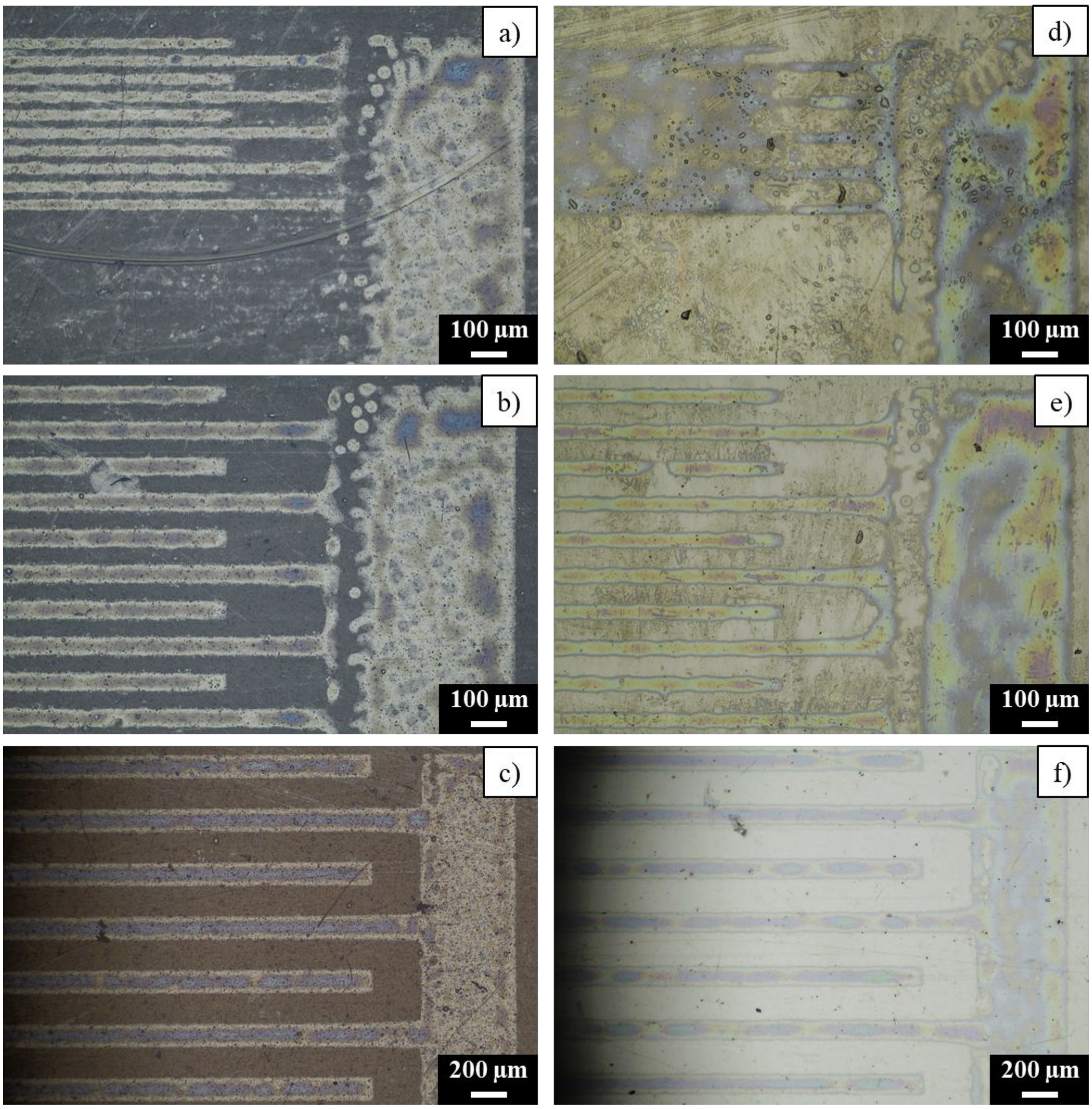

Figure S3. Optical microscope images of SV4 STAB prints with different finger widths: a) 20 µm, b) 40 µm, and c) 120 µm on Flextrace, and d) 20 µm, e) 40 µm, and f) 120 µm on Melinex.

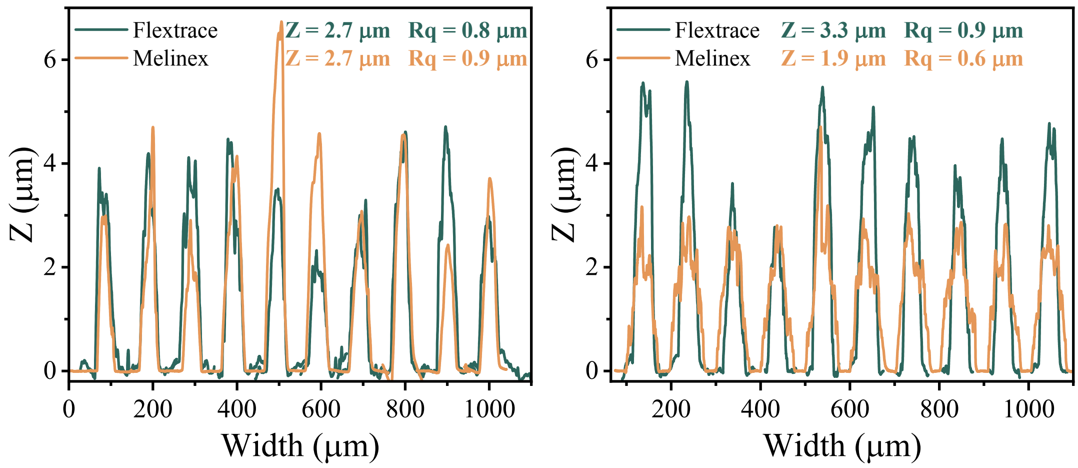

Figure S4. Finger thickness of AGFA SI-P2000 (left) and SCAG-004P (right) patterns printed on Flextrace and Melinex.

Table S1. Thickness and roughness of 40 µm finger width samples.

Ink	Thickness (µm)	Roughness (µm)
AGFA SI-P2000 F	2.7	0.8
AGFA SI-P2000 M	2.7	0.9
DuPont PE827 F	4.0	1.2
DuPont PE827 M	2.7	0.8
SCAG-004P F	3.3	0.9
SCAG-004P M	1.9	0.6
SV4 STAB F	-	-
SV4 STAB M	0.14	0.04
SEBS/15%rGO F 1x	0.7	0.4
SEBS/15%rGO F 3x	3.0	1.0
SEBS/45%NrGO F 1x	1.08	0.42
SEBS/45%NrGO F 3x	3.0	1.0
